# Decompensation of Cirrhosis: An Unusual Cause

**DOI:** 10.7759/cureus.24851

**Published:** 2022-05-09

**Authors:** Omkar S Rudra, Sherna Menezes, Aabha Nagral

**Affiliations:** 1 Critical Care Medicine, Jaslok Hospital and Research Centre, Mumbai, IND; 2 Gastroenterology, Jaslok Hospital and Research Centre, Mumbai, IND; 3 Gastroenterology, Apollo Hospital, Navi Mumbai, IND

**Keywords:** salmonella paratyphi b, hcv cirrhosis, non-typhoidal salmonella (nts), recurrent urinary tract infections, nephrolithiasis

## Abstract

We describe a case of a 69-year-old woman, a known case of compensated Hepatitis C cirrhosis, presenting with recurrent urinary tract infection (UTI) secondary to* Salmonella Paratyphi B*. She had recently tested positive for* Salmonella Paratyphi *bacteremia associated with UTI, managed with antibiotics. Two weeks later, she presented with fever and burning micturition with signs of abdominal distension and pedal edema suggestive of decompensation of cirrhosis. She had repeat positive urine cultures for Salmonella and was managed with broad-spectrum antibiotics, surgical stone removal, and supportive measures leading to resolution of symptoms and ascites. She was administered a prolonged antibiotic course on discharge for chronic shedding of Salmonella in the urine.

UTIs are a common cause of decompensation in immunosuppressed states like liver cirrhosis, and rare infections like salmonella need to be identified and managed appropriately with sensitive antibiotics and the right duration of treatment.

## Introduction

Salmonella bacteriuria is rare and requires a high index of suspicion for diagnosis [1]. Prolonged shedding of Salmonella in the urine can be seen after a urinary tract infection (UTI) in patients with nephrolithiasis or urinary tract abnormalities, mandating long-term antibiotic therapy with a surgical opinion for source control [1]. Bacterial infections, including UTIs, occur more frequently in cirrhotic patients due to their immunocompromised state and, if not adequately and promptly treated, can lead to rapid decompensation in patients with cirrhosis [2,3].

## Case presentation

A 69-year-old woman with type 2 diabetes mellitus and compensated cirrhosis with portal hypertension secondary to Hepatitis C was admitted for complaints of fever and 10 to 12 episodes of loose stools over three to four days. There was no history of abdominal pain or flank pain. In the past, she had received treatment for Hepatitis C with directly acting antivirals following, which sustained viral response (SVR 12) was achieved. She was compliant with her treatment for diabetes mellitus. Her medication history included insulin, propranolol, and rifaximin.

Upon presentation, her vitals were significant, with a temperature of 100.4°F with a regular pulse of 104/minute and blood pressure of 168/63 mmHg. There were no signs of hepatic encephalopathy. Her physical examination was unremarkable. The laboratory work-up at the time revealed hemoglobin of 9 g/dl, with a total leucocyte count of 5,560 cells/cumm and platelet count of 68,000/cumm. Liver function tests were within normal limits, with an international normalized ratio (INR) of 1.28 and a calculated model for end-stage liver disease (MELD-Na) of 12. Renal function and stool routine examinations were normal, while urine evaluation was suggestive of a UTI with >200 pus cells/hpf. Pending blood, urine, and stool culture and sensitivity reports, she was started on a combination of cefoperazone with sulbactam. She underwent ultrasonography (USG) of the abdomen, which showed a 15 mm calculus at the pelvic-ureteric junction on the right side with mild hydronephrosis and moderate dilation of the renal pelvis. It also showed minimal ascites with cirrhosis, splenomegaly, and a dilated main portal vein. She was advised of a surgical opinion for stone removal, which the patient refused. The stool cultures were negative, but urine and blood cultures grew Salmonella Paratyphi B [heavy growth of > 10^5^ Colony Forming Units (CFU)] which was sensitive to the combination, and she was thus continued on it for seven days. She was subsequently discharged.

**Figure 1:**
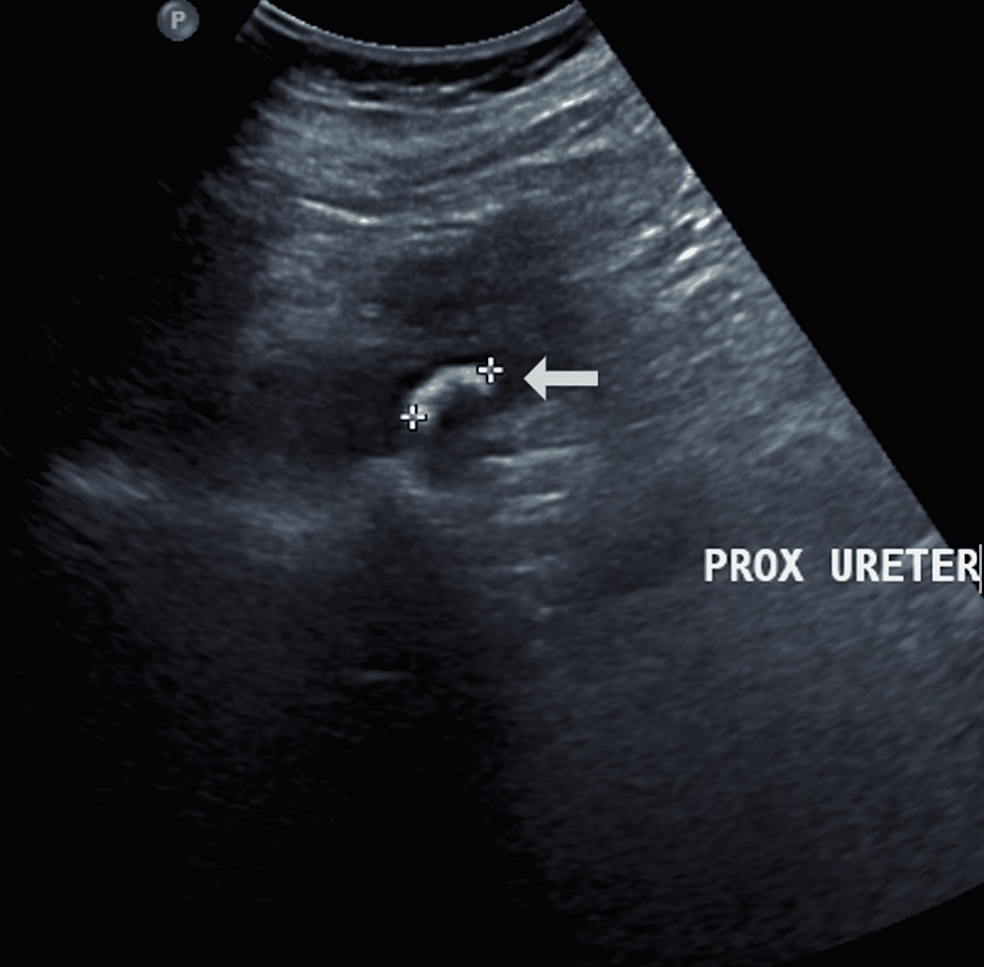
Ultrasonography of the abdomen showed a 15 mm calculus in the proximal right ureter at the pelvic-ureteric junction

**Figure 2:**
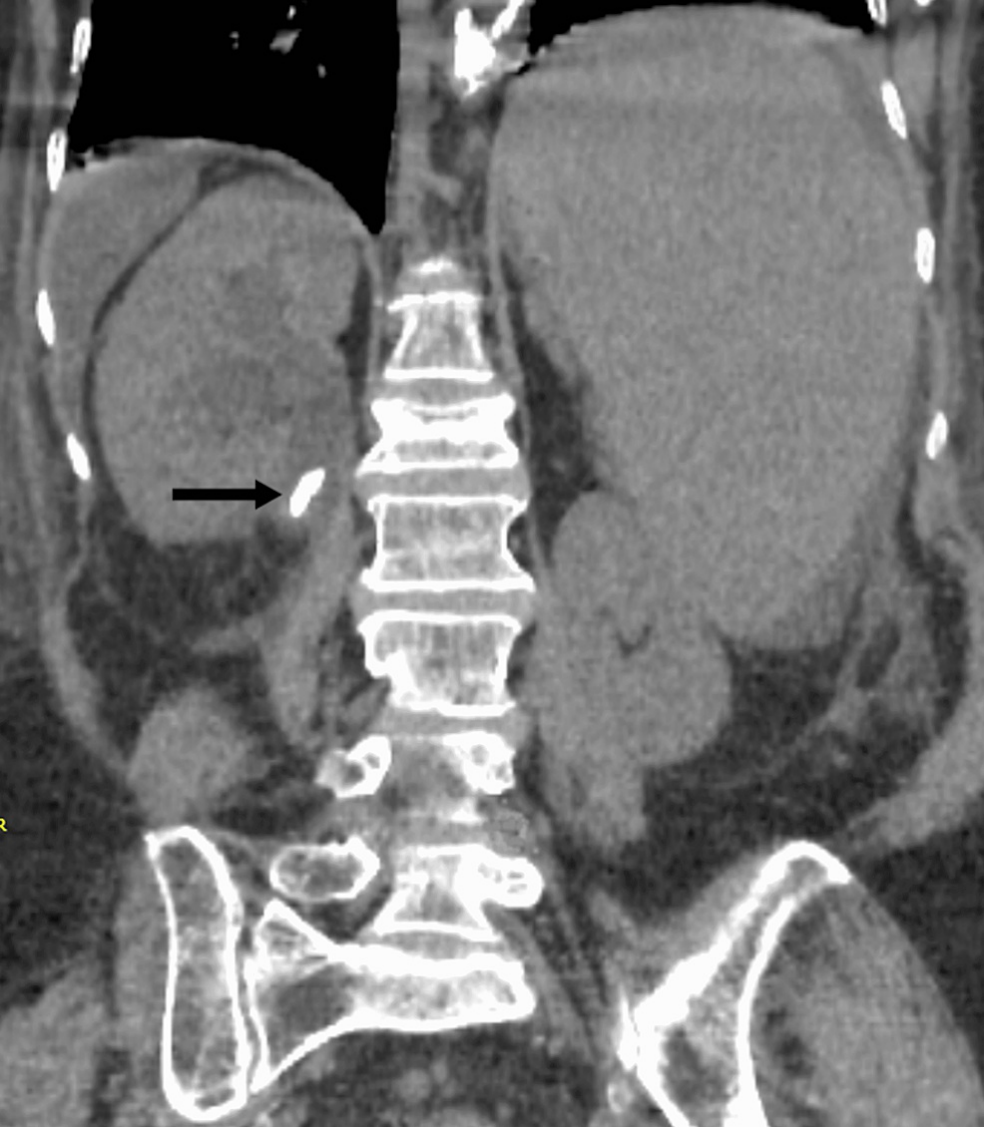
Non-contrast CT scan showing calcific calculus in right pelvic-ureteric junction measuring 15mm x 5mm with right hydronephrosis CT: computed tomography

She returned two weeks later with burning micturition and pedal edema for the past seven days with intermittent episodes of fever. Her vitals were unremarkable, with the physical examination demonstrating bilateral pitting pedal edema and a distended, non-tender abdomen suggestive of moderate free fluid. She showed no signs of hepatic encephalopathy. The remainder of the examination was unremarkable. In this admission, the laboratory work-up showed a haemoglobin of 9.5 g/dL with a hematocrit of 29, a total leucocyte count of 6,160 cells/cumm, and a platelet count of 53,000/cumm. Her renal function revealed a serum creatinine of 0.7 mg/dl, with serum electrolytes being within normal limits. Apart from a slightly elevated aspartate aminotransferase (AST) of 87 IU/L, INR of 1.36, and low serum albumin of 2.9 g/dL, the liver function tests were unremarkable (MELD-Na 14). Her random blood glucose was 159 mg/dL. The repeat urinalysis on admission revealed >200 pus cells, 40-50 red blood cells, trace albumin, and ketones with the culture suggestive of Salmonella Paratyphi B (heavy growth > 10⁵ CFU). She was subjected to ultrasonography of the abdomen, which revealed a 15 mm calculus at the pelvic-ureteric junction on the right side associated with mild hydronephrosis and moderate dilation of the renal pelvis (Figure 1). It also showed gross ascites with cirrhosis, splenomegaly, and dilatation of the main portal vein. These findings were confirmed on a non-contrast computed tomography scan (Figure 2). USG-guided diagnostic and therapeutic abdominal paracentesis of 3.8 liters was done, which revealed a cell count of 180 cells/cumm with 20% neutrophils, 80% lymphocytes, no red blood cells, and a high serum-ascites albumin gradient secondary to portal hypertension. Empirically, piperacillin-tazobactam was initiated, since she had already received the combination of cefoperazone and sulbactam. She was also started on diuretics on admission. A urology opinion was sought for the management of the right renal calculus and hydronephrosis, and subsequently, she underwent retrograde intrarenal surgery for removal of the calculus, and a double J-stent was placed. She was advised Trimethoprim-Sulfamethoxazole for three weeks based on culture reports, given chronic shedding of Salmonella Paratyphi B in urine upon her discharge.

 

She followed up after three weeks of antibiotic treatment, and her ascites and pedal edema showed significant reduction. Her liver function tests also showed improvement, with AST level falling to 58 IU/L and serum albumin increasing to 3.8 g/dL. Her repeat urinalysis and cultures were unremarkable, with complete resolution of symptoms. She also underwent double J-stent removal.

## Discussion

Salmonella infections of the urinary tract are uncommon and require a high index of suspicion for diagnosis. Salmonella Paratyphi B is a gram-negative bacteria transmitted via the fecal-oral route, which can invade the bloodstream and cause paratyphoid fever. Salmonella infections most commonly present as acute gastroenteritis and occasionally lead to bacteremia, an asymptomatic carrier state, or focal infections. In large retrospective reviews in the United States, non-typhoidal salmonella (NTS) UTI has been found to comprise only 0.63% of all salmonella infections [4]. The incidence of NTS UTI is less than 1% of cases, even in endemic areas like India [5]. 

NTS has been postulated to enter the urinary tract either via direct invasion of the tract following salmonella enteritis or hematogenous. Systemic risk factors for NTS UTI include recent Salmonella gastroenteritis, and patients with conditions causing immune suppression, such as those with diabetes mellitus, chronic kidney disease on hemodialysis, liver disease, Human Immunodeficiency Virus (HIV)/Acquired Immunodeficiency Syndrome (AIDS), rheumatological diseases, patients of renal transplant or solid tumors [6,7]. The short urethra in women has been considered a local risk factor for UTI, while for men, partial obstruction causing stasis is said to play a role in the development [8]. Other genito-urinary conditions predisposing to NTS UTI include prior surgery, stones, strictures, duplicating collection system, indwelling urinary catheter, enteric fistula, urinary stasis due to pregnancy, vesicoureteral reflux or enlarged prostate, and these conditions must be considered in the workup of patients who are found to have Salmonella UTIs [1,6,7,9]. Our patient was predisposed to such an infection, most likely due to a combination of nephrolithiasis and the immunosuppressed state seen in patients with cirrhosis. The immunosuppressive nature of the patient’s condition led to the possible hematogenous spread of the Salmonella infection.

Symptoms of Salmonella UTI do not differ from the symptoms of UTI caused by other gram-negative organisms. Complications of NTS UTI include pyelonephritis, abscesses, recurrence, and chronic bacteriuria [6,9]. Treatment of NTS is difficult owing to these complications and requires prolonged antibiotic therapy based on sensitivity reports [10-12]. In a study of 19 patients with UTI due to NTS conducted by Tena et al., 11 patients (57.8%) cleared the infection after 3 weeks or more of the antibiotic course, while 6 patients (31.5%) required more than 3 weeks of treatment. 2 patients (one with systemic lupus erythematosus and the other with urolithiasis) had recurrences despite prolonged therapy of 3.5 and 5 weeks, respectively [1]. As in our case, such patients with stones/urological abnormalities often require surgical removal of the stones/surgical correction of the abnormality along with antibiotic therapy to eliminate the carrier state [13]. Our patient developed recurrent infections as the source of infection persisted, and the first infection did not receive a prolonged antibiotic course.

## Conclusions

Patients with underlying chronic illnesses like liver disease are maintained a fragile balance, with common infections such as UTIs causing rapid decompensation. While NTS UTI is uncommon, its diagnosis is important since treatment requires a prolonged course of sensitivity-guided antibiotics, unlike other routinely diagnosed causes of UTI. A positive urine culture must also prompt an evaluation for local causative factors. Follow-up with repeat urine cultures is necessary since recurrences can be seen despite prolonged antimicrobial therapy.
